# Epiploic Appendagitis: A Rare Cause of Acute Abdominal Pain

**DOI:** 10.31662/jmaj.2022-0199

**Published:** 2023-03-06

**Authors:** Masahiro Ito, Toshiki Sato, Tsuneo Nagai

**Affiliations:** 1 Department of Internal Medicine, Nagaoka-Nishi Hospital, Niigata, Japan; 2 Nagaoka Sutoku University, Niigata, Japan

**Keywords:** epiploic appendagitis, computed tomography, acute abdomen

A previously healthy 48-year-old man presented with sudden-onset lower left abdominal pain, which persisted for 3 days. On physical examination, he had left lower quadrant tenderness. Laboratory results showed an increased white blood cell count (9.8 × 1000/μl) and increased C-reactive protein (1.8 mg/dL). Abdominal computed tomography (CT) revealed a fat-density ovoid structure with a high-density rim adjacent to the sigmoid colon and surrounding inflammatory fat, which was consistent with epiploic appendagitis (Figure 1). The patient was treated with a nonsteroidal anti-inflammatory drug and his pain resolved after 1 week. Epiploic appendagitis has nonspecific clinical findings ^(1)^, and abdominal CT is of critical importance to properly diagnose this disease and distinguish it from other surgical emergencies responsible for acute abdomen ^(2)^. This condition is basically a self-limiting disease; however, laparoscopic appendage excision may be required for recurrent cases ^(1)^. Epiploic appendagitis is a rare condition but should be considered in patients with acute abdominal pain.

**Figure 1.**
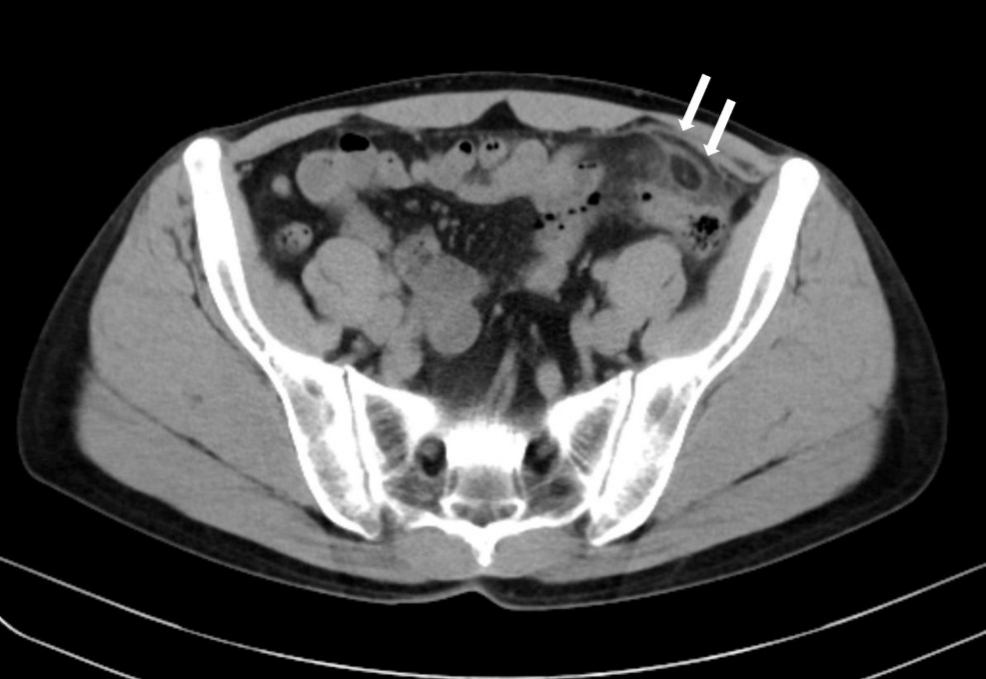
Computed tomography revealed the presence of a fat-density ovoid structure with a high-density rim adjacent to the sigmoid colon and surrounding inflammatory fat, suggesting epiploic appendagitis.

## Article Information

### Conflicts of Interest

None

### Author Contributions

MI wrote the first draft and managed the submission processes. All authors (1) made substantial contributions to the study concept and data analysis/interpretation; (2) drafted the manuscript and revised it critically for important intellectual content; (3) approved the final version of the manuscript to be published; and (4) agreed to be accountable for all aspects of the work.

### Informed Consent

We have obtained informed consent for the publication of this case report.
